# Does including machine learning predictions in ALS clinical trial analysis improve statistical power?

**DOI:** 10.1002/acn3.51140

**Published:** 2020-08-30

**Authors:** Nina Zhou, Paul Manser

**Affiliations:** ^1^ Department of Biostatistics University of Michigan Ann Arbor Michigan USA; ^2^ Department of Biostatistics Genentech, Inc. South San Francisco California USA

## Abstract

**Objective:**

Amyotrophic lateral sclerosis (ALS) is a neurodegenerative disease which leads to progressive muscle weakness and eventually death. The increasing availability of large ALS clinical trial datasets have generated much interest in developing predictive models for disease progression. However, the utility of predictive modeling on clinical trial analysis has not been thoroughly evaluated.

**Methods:**

We evaluated a predictive modeling approach for ALS disease progression measured by ALSFRS‐R using the PRO‐ACT database and validated our findings in a novel test set from a former clinical trial. We examined clinical trial scenarios where model predictions could improve statistical power for detecting treatment effects with simulated clinical trials.

**Results:**

Models constructed with imputed PRO‐ACT data have better external validation results than those fitted with complete observations. When fitted with imputed data, super learner (*R* ^2^ = 0.71, MSPE = 19.7) and random forest (*R* ^2^ = 0.70, MSPE = 19.6) have similar performance in the external validation and slightly outperform the linear mixed effects model (*R* ^2^ = 0.69, MSPE = 20.5). Simulation studies suggest including machine learning predictions as a covariate in the analysis model of a 12‐month clinical study can increase the trial's effective sample size by 16% when there is a hypothetical treatment effect of 25% reduction in ALSFRS‐R mean rate of change.

**Interpretation:**

Predictive modeling approaches for ALSFRS‐R are able to explain a moderate amount of variability in longitudinal change, which is improved by robust missing data handling for baseline characteristics. Including ALSFRS‐R post‐baseline model prediction results as a covariate in the model for primary analysis may increase power under moderate treatment effects.

## Introduction

Amyotrophic lateral sclerosis (ALS) is a fatal neurodegenerative disease characterized by degeneration of motor neurons which leads to progressive muscle weakness and paralysis. Disease progression rates vary with death typically occurring within 3–5 years from symptom onset; however, patients may live decades with the disease. Although existing drugs^1^, ^2^ are approved for ALS treatment, there remains a significant unmet need to meaningfully reduce functional decline and prolong survival.

One of the challenges for conducting ALS clinical trials is the substantial heterogeneity in clinical presentation and rate of progression.^3^ One approach to address this heterogeneity recently employed in a successful clinical trial of edaravone was to use a highly stringent^4^ set of inclusion/exclusion criteria to select a targeted ALS patient population. Despite this advance, more sophisticated model‐based methods may help to even better inform trial conduct and analysis approaches to increase efficiency and sensitivity of ALS clinical trials, potentially in a broader patient population. The availability of large ALS data sets has created opportunities for the development of such predictive models.

Predictive models have been developed to evaluate Revised Amyotrophic Lateral Sclerosis Functional Rating Scale (ALSFRS‐R)^5^ scores and overall survival using clinical trial and ALS registry data. Predictive models trained from clinical trial data^6^, ^7^ have been used to inform patient enrichment, stratify randomization, and perform covariate adjustment, with the aim of reducing the required sample size to achieve a desired level of power. Models fit to ALS registry data^8^ have also been developed for managing patient care and predicting long‐term survival outcomes. The level of accuracy and precision of these methods, along with their clinical utility, requires careful consideration when determining appropriate use. It is imperative to ensure that clinical trials are adequately powered, interpreted correctly and transparently, and that any predictive model enhances these aims.

The aims of this paper are twofold: (1) to take a critical look at predictive modeling for ALSFRS‐R using models built on data from the Pooled Resource Open‐Access ALS Clinical Trials (PRO‐ACT) database^9^; and (2) to identify ALS clinical trial scenarios where predictive modeling approaches do and do not make a meaningful difference in the analysis of clinical trials simulated from an independent test data set.

## Materials and Methods

### Training data

**Figure 1:**
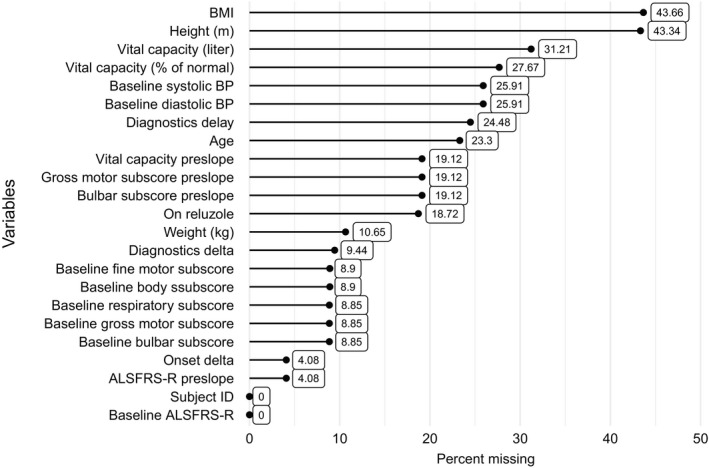
Missing pattern for demographic predictor variables in PRO‐ACT.

The PRO‐ACT database, is a comprehensive dataset integrating 23 clinical trials, and has been a valuable resource for building ALS progression prediction models in previous studies.^6^, ^7^, ^9^, ^10^ While the full PRO‐ACT database contains over 10,700 patient records, our analysis focuses on a subset of 3160 patients with an available baseline ALSFRS‐R score and at least one post‐baseline ALSFRS‐R. Patients were allowed to have missing values for other baseline covariates. Separate models were fit to a data set including only patients with complete baseline records of possible predictors (Figure 1) without missing values (n = 552) and a second set of models with an imputed baseline data set to enable inclusion of all 3160 patients.

### Test data

The test data came from the MITOTARGETPhase II/III double blind, placebo‐controlled trial of olesoxime. The study enrolled 512 patients with El Escorial probable or definite ALS.^11^ 15 centers across five European countries. Patients were observed over a 18‐month planned treatment duration and all were required to be on a stable dose of 50 mg riluzole taken twice a day for at least 1 month prior to enrollment. Subjects were required to have SVC ≥70% at screening with time of symptom onset between 6 and 36 months. All covariates used for modeling in the training data set were available in the test data set and were considered for model validation. Only three patients in the olesoxime data had any missing values at baseline. The final validation set consisted of the 509 patients with complete baseline records.

### Missing data handling

Missing data are a critical issue for PRO‐ACT data utilization. To enrich the available patient pool for predictive model fitting, we considered imputing baseline covariates with an iterative nonparametric imputation method. First, we analyzed the missingness pattern with visualization plots and used Little's MCAR test^12^ to assess if missing completely at random (MCAR) is a valid missingness mechanism for PRO‐ACT data. Little's MCAR test suggested that the missing data are not MCAR (*P* < 0.001). We then assumed that the missingness can be modeled from observed values and follows a MAR pattern. Lastly, we imputed the missing values in each baseline covariate using missForest,^13^ an iterative imputation approach for mixed‐type data utilizing the random forest (RF) algorithm. We utilized missForest as our imputation method to create a single complete data for constructing ALSFRS‐R prediction models. The method has better performance in imputation accuracy than popular approaches including multiple imputation.^13^, ^14^ After handling missing data, a sensitivity analysis was performed to compare the predictive models constructed by the imputed and complete case data sets.

### Model covariates and outcome

Predictors included in the models were similar to those used in previous modeling efforts using the PRO‐ACT database.^7^ We considered 23 variables as the candidate covariates for predicting ALSFRS‐R total score. Number of days on study was used to characterize time from baseline ALSFRS‐R assessment. Among the remaining variables, 11 of them are patient demographics recorded at baseline. Six total and sub baseline functional scores are also selected. Finally, slopes (referred to as pre‐slopes) for four of the baseline functional scores were derived and considered as candidate predictors. The preslopes for a given functional score were calculated using the following formula:

$$\frac{\text{Max possible score}‐\text{Baseline score}}{\text{Time from symptom onset to baseline}},$$

and included in our set of potential covariates.

### Candidate models

We compared the performance of three models, each built twice using the imputed and complete case PRO‐ACT datasets. The three candidate models were RF,^15^ super learner (SL),^16^ and a linear mixed effects model with random intercepts (LME).^17^ Both machine learning models were constructed using days from the baseline and cross‐sectional baseline covariates as predictors.

Tuning was considered for both machine learning models to control for overfitting. For RF, we fixed the number of trees to be 500 and performed stepwise tuning for the number of variables per node with respect to out‐of‐bag error. Based on results of 10‐fold cross validation, the RF model was optimized by randomly sampling eight candidate variables per split. Weights of the base learners in SL were also tuned with 10‐fold cross‐validation using Out‐of‐Bag error. We considered *Xgboost*, linear regression, RF, and generalized LME model with ENet regularization as the four candidate learners for the SL model. The cross‐validation results on imputed PRO‐ACT suggested that RF and xgboost should be considered as base learners, where RF has a dominating weight of 0.98.

The predictors with highest variable importance in RF were included in the LME to avoid multicollinearity. The LME uses linear predictors without interactions and a random intercept as a simple, parsimonious model. Based on the importance ranking from the RF model, days on study, baseline FRS, FRS preslope, and baseline body subscores were the most salient predictors in decreasing the node impurity (Gini Index). Since baseline FRS and body subscore are highly correlated, the resulting parsimonious LME model contained days on study, baseline FRS, FRS preslope, Age, and time from onset to baseline:

$$\text{ALSFRS}‐R_{i,j}=\beta_{0}+\beta_{1}{\text{ALSFRS}}_{i,0}+\beta_{2}{\text{Preslope}}_{i}+\beta_{3}{\text{Days on study}}_{i,j}+\beta_{4}{\text{Age}}_{i}+\beta_{5}\text{Onset Delta}+b_{i}+\varepsilon_{i,j},$$

where ALSFRS*_i_*_,0_ is the baseline FRS score, $b_{i}∼N\left(0,\sigma_{b}^{2}\right)$ is the random intercept for patient *i*, and $\varepsilon_{i,j}$ is the random error.

### Model validation and comparison

The predictive power of the three candidate models was compared in both the PRO‐ACT and olesoxime data. First, cross‐validation in the PRO‐ACT data was conducted using a randomly selected 25% subset as a test set and the remaining 75% as training data. Final models were trained using all available PRO‐ACT data. To assess model generalizability, we calculated the prediction accuracy of each model in the olesoxime data.

To compare model fits, we considered recording mean squared prediction error (MSPE) and using simple linear regression to regress the observed ALSFRS‐R on the predicted ALSFRS‐R. *R* ^2^ of the regression model was used to measure the variation of the observed explained by the predicted and the intercept was used to measure bias. A similar approach was used to predict change from baseline, rather than ALSFRS‐R total score, as this is generally of more interest in clinical trial design.

### Simulation study design

Accurate ALSFRS‐R predictions can serve as a dimension reduction technique to control for high‐dimensional patient characteristics and synthetic controls before randomization. We conducted simulation designs focusing on examining the increase in statistical power when including model predictions as a covariate in the treatment model for the primary analysis.

The simulated patient records were generated by randomly sampling the olesoxime data without replacement. For the treatment arm, we introduced a constant treatment effect to the original sampled record by reducing their ALSFRS‐R change from baseline by a certain percentage. Therefore, the ALSFRS‐R score at all post‐baseline times *j* for patient *i* in the treatment arm will be updated into:

$$Y_{i,j}^{\text{trt}}=\left(Y_{\mathrm{ij}}‐Y_{i0}\right)\times E+Y_{i0},$$

where *Y_i_*_,_ *_j_* and *Y_i_*_0_ are the observed ALSFRS‐R for patient *i* at baseline and time *j*, respectively, and *E* is the introduced shrinkage percentage. Study settings with different sample sizes, study durations and treatment effects were considered, each had 10,000 replicates randomly assigning patients to active or placebo in 1:1 ratio. We considered 6‐, 12‐, and 18‐month study durations with sample sizes of *n* = 120, 160, 200, …, 360. Type one error was examined for the scenario with no treatment effect (*E* = 0%). A smaller effect of *E* = 25% and larger effect of *E* = 40% were also considered. No treatment effect was introduced on patient survival.

Treatment effects were estimated with seven different LME models (Table 1). All models included baseline ALSFRS‐R, categorical visit terms and treatment visit interactions as covariates. RF, LME, and SL also controlled for predictions as time‐varying covariates. The single best predictor model included ALSFRS‐R preslope as an additional baseline covariate.^7^ The standard baseline covariates model controlled for standard stratification factors in ALS clinical trials: riluzole use and site of onset. The No extra covariates model had no extra demographic variables. The full model including RF predictions, preslope, and standard baseline incorporated all covariates in RF, single best predictor, and standard baseline covariates. To examine the sensitivity of treatment effect detection for each model, we plotted power curves (with type I error controlled at *α* = 0.05 level) and win rate curves with different scenarios and sample sizes. The win rate for a model is defined as the chance of having the smallest *P*‐value amongst all models.

**Table 1:** Candidate linear mixed effect models in the simulation studies to measure treatment effects.

Models	Intercept and baseline FRS	Extra baseline variables	Time & time treatment interactions
Random forest predictions	$Y_{\mathrm{ij}}‐Y_{i0}=\beta_{0}+\beta_{1}Y_{i0}+$	$\beta_{2}{\text{RFpred}}_{\mathrm{ij}}$	$+\Sigma_{t=1}^{J}\alpha_{t}I\left(t=j\right)+\Sigma_{t=1}^{J}\gamma_{t}I\left(t=j\right)\times I\left(\text{Trt}\right)+\varepsilon_{\mathrm{ij}}$
Linear mixed effects model predictions		$\beta_{2}{\text{LMMpred}}_{\mathrm{ij}}$	
Super learner predictions		$\beta_{2}{\text{SLpred}}_{\mathrm{ij}}$	
Single best predictor		$\beta_{2}{\text{preslope}}_{i}$	
Standard baseline covariates		$\beta_{2}{\text{SiteOnset}}_{i}+\beta_{3}\text{El}$	
No extra covariates		–	
Full model including random forest predictions, preslope, and standard baseline		$\beta_{2}{\text{RFpred}}_{\mathrm{ij}}+\beta_{2}{\text{preslope}}_{i}+\beta_{4}{\text{SiteOnset}}_{i}+\beta_{5}\text{El}$	

For 6‐month studies, we consider follow‐up time to be baseline, Month 1, Month 2, Month 3, and Month 6. For 12‐month studies, we consider follow‐up time to be baseline, Month 1, Month 6, and Month 12. For 18‐month studies, we consider follow‐up time to be baseline, Month 1, Month 6, Month 12, and Month 18. The following models are estimated with unstructured correlation structure.

### Data sharing

Qualified researchers may request access to individual patient level data through the Vivli Center for Global Clinical Research Data (https://vivli.org/) clinical study data request platform (www.clinicalstudydatarequest.com). Further details on Roche's criteria for eligible studies are available here (https://clinicalstudydatarequest.com/Study‐Sponsors/Study‐Sponsors‐Roche.aspx). For further details on Roche's Global Policy on the Sharing of Clinical Information and how to request access to related clinical study documents, see here (https://www.roche.com/research_and_development/who_we_are_how_we_work/clinical_trials/our_commitment_to_data_sharing.htm).

## Results

### Comparison of demographic characteristics

The baseline clinical and demographic characteristics of the complete and imputed PRO‐ACT and olesoxime data are described in Figure S1. The patients in the three data sets are highly comparable since most of the variables had similar distributions. The majority of the ALS patients are in their 50 and 60 sec, with average baseline ALSFRS‐R ranging from 37.77 to 38.63. Patients in the imputed PRO‐ACT (547 days) and olesoxime (522 days) data share similar time difference from symptom onset to baseline, while patients in the complete data (684 days) had larger time differences. Moreover, the imputed PRO‐ACT data includes a broader population with a wider range of vital capacity.

Among the patients with observed ALSFRS‐R, only 552 had complete data of the 23 demographic variables listed. The missingness patterns for candidate predictors are illustrated in Figure 1. BMI and height were the variables with the highest missing proportion (over 40%). Most remaining variables were missing less than 25% of observations.

### Model prediction results

The three models performed similarly on the cross‐validated PRO‐ACT data (Table 2), with SL having the smallest cross‐validated MSPE 26.66 followed by RF (26.68) and LME model (27.34). The *R* ^2^ values for all models were around 0.66. None of the models have a significant bias in prediction for the imputed data set. Sensitivity analysis suggests that missing data imputation improved model generalizability. Models fitted on complete data have slightly better performance in internal cross‐validation but worse performance in external validation.

**Table 2:** Cross‐validation results for imputation after separating the PRO‐ACT database into training (75% data) and testing (25% data) sets.

Model	Complete			Imputed		
	*R* ^2^	Intercept (*P*‐value)	MSPE	*R* ^2^	Intercept (*P*‐value)	MSPE
Random forest	0.674	−2.182 (0.022)	18.046	0.668	0.038 (0.887)	26.685
Super learner	0.677	−2.264 (0.017)	17.900	0.668	−0.081 (0.761)	26.668
Linear mixed effects model with random intercepts	0.675	−0.655 (0.474)	17.760	0.660	0.301 (0.265)	27.348

MSPE, mean squared prediction error.

**Figure 2:**
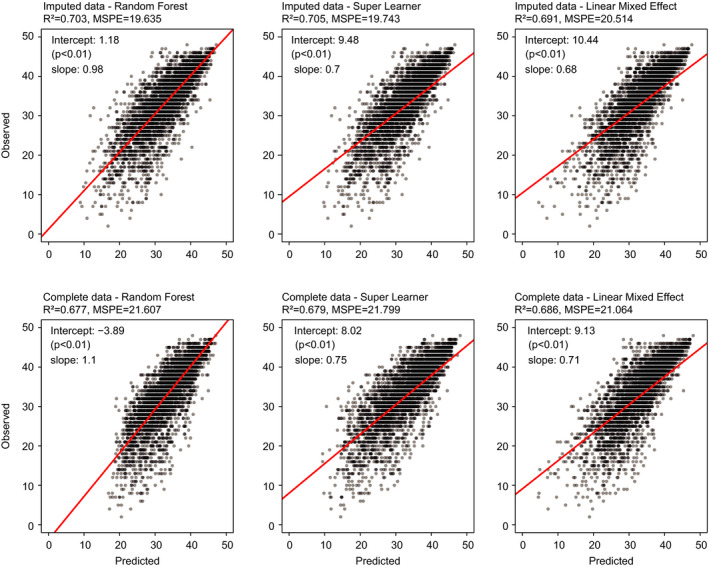
Model prediction results on olesoxime data at all time points. The scatterplots are constructed with linear models regressing observed ALSFRS‐R on model predictions. Mean squared prediction error (MSPE) and *R* ^2^ are reported. Intercepts and slopes of the linear models indicate the prediction bias of the corresponding models.

Results of the prediction models in the external validation dataset are visualized in Figure 2 by plotting the observed data against model predictions. *R* ^2^ was approximately 0.7 for all three models (ranging from 0.677 to 0.705). RF and SL both marginally outperformed the LME with a marginal advantage in MSPE (0.9 points) and *R* ^2^ (1%) with RF having the smallest bias in both cross‐validation and external validation.

**Figure 3:**
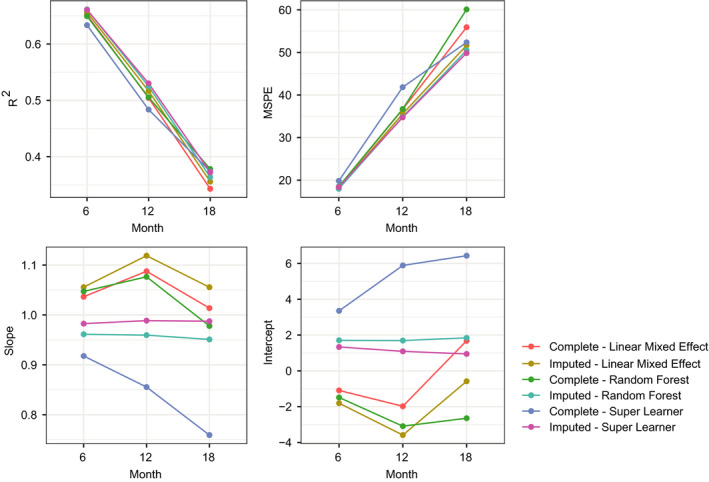
Prediction results for olesoxime data stratified by month. Each dot shows the mean squared prediction error (MSPE) of the predictions or *R* ^2^, slope and intercept of a linear model regressing actual ALSFRS‐R on predicted values.

When looking at model performance as a function of time on study, Figure 3 shows that in terms of *R* ^2^ and MSPE, predictive performance for all models becomes poorer farther from baseline. In terms of bias, RF and SL had the least amount of bias, which was consistent among different timepoints.

### Simulation studies

**Figure 4:**
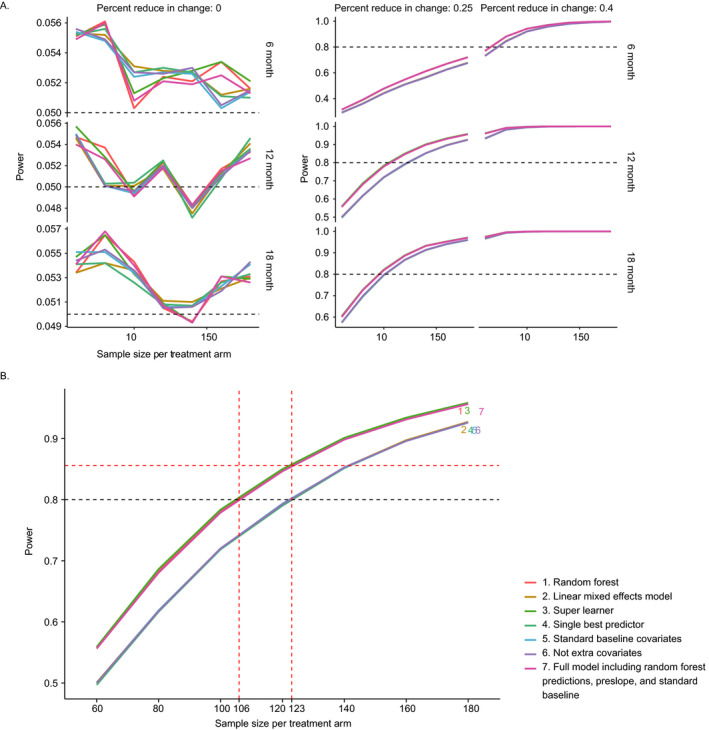
(A) Power analysis from simulation studies. The columns represent different hypothetical treatment effects 0%, 25%, and 40%. The rows denote study duration from 6 to 18 months. In each plot, the *y*‐axis characterizes power and x‐axis are the sample sizes per treatment arm from 60 to 180. The seven models considered in the plots are specified in Table 1, where the first 3 models are including to ALSFRS‐R predictions as one of the covariates. (B) Power analysis from a 12‐month simulation study with hypothetical treatment effect as 25%. RF, SL and the full model including random forest predictions, preslope, and standard baseline showed a significant power benefit than the other models. When performing a clinical trial with a sample size of 106 patients per arm, these models have increased the effective sample size by 17 patients per each arm comparing to other models.

Figure 4A shows the power curves for the seven models under different simulation settings. Columns represent treatment effects from no (*E* = 0%) to substantial (*E* = 40%) treatment effect and the rows represent different study lengths. The 3 figures in the first column suggest that type one error was controlled at around 0.05 under different model specifications and study lengths. The right‐most column indicates that when the treatment effect is large, the power of the study is well above 0.8 under any of the considered sample sizes. The medium column shows that a 6‐month study is not able to achieve 0.8 power under the small treatment effect. All models have similar performance in detecting the treatment effect in an 18‐month study.

As illustrated in Figure 4B, for simulated 12‐month studies with small effect size, RF, SL, and full model including RF predictions, preslope, and standard baseline models have higher power compared to LME, single best predictor, standard baseline covariates, and no extra covariates. When considering an ALS study of 212 patients, the models including RF or SL predictions can increase the effective sample size by approximately 34 patients or 17 patients per each arm, which is 16% of the original sample size. However, in most settings, a meaningful difference among the models was not observed.

**Figure 5:**
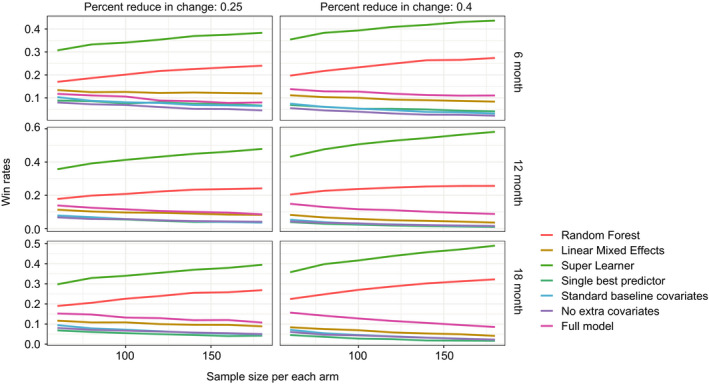
Win percentage plots for smallest *P*‐values regarding the treatment time interaction coefficient in different models from the simulated clinical trial analysis. The two columns represent different levels of treatment effects. The rows represent simulation study length. Each small plot records different models' chance of winning (has the smallest *P*‐value for the treatment and last visit time interaction) across possible sample size. Ten thousand simulated datasets were generated under each treatment effect, study length, and sample size combination. SL, and RF models had the highest and second highest probability to produce the smallest *P*‐value under all scenarios, respectively.

To assess relative model performance independent of a nominal significance level (e.g. *α* = 0.05), we ranked the 7 models by their *P*‐values for each simulation iteration. The win percentage plot was included in Figure 5. SL had the smallest *P*‐values for 30–60% of the times and RF had the smallest *P*‐values for 20–30% of the times across all scenarios in our simulation.

## Discussion

Effectively aggregating data across clinical trials can be challenging for constructing predictive models for ALSFRS‐R, because of differing degrees of missing data between trials. In this paper, we characterized the missingness pattern in the PRO‐ACT database, used an established imputation approach to address the missing data issue, dramatically increased the available sample size, and showed that our treatment of missing data improved model fit and reduced prediction bias in the validation data. For aggregated clinical trials data like PRO‐ACT, where missingness patterns of baseline demographic variables are typically not informative, imputation methods can effectively handle variables with moderate missingness and are a practical solution for making the most of available data.

There is a difference between missForest and multiple imputation (MI) methods in terms of the number of imputed data sets generated. missForest's intended use is for generating a single dataset optimized for imputation accuracy whereas MI's intended use is for leveraging multiple imputed datasets to characterize uncertainty of imputed values when conducting statistical inference. In our use case of building a predictive model, missForest is the preferable method because it simplifies predictive model building by using a single imputed data set with the expectation of better performance relative to MI.^14^ missForest is not designed to generate multiple imputed data sets to characterize uncertainty of imputed values when conducting statistical inference, like MI.

Our results suggest that RF was the best prediction model among the three tested models in terms of bias, variance, and generalizability to the external validation data set. We observed similar, if slightly higher, *R* ^2^ values relative to what has been reported previously using RF models with the PRO‐ACT data. Notably, SL, a more complex ensemble‐based method, could not meaningfully improve upon the performance of RF. The prediction results on the olesoxime data were indistinguishable between the two models. Hence, RF is the preferable prediction model due to its computational efficiency and interpretability.

We also assessed the improvement of statistical power when including model prediction as a covariate in the treatment model for the primary analysis. Two predictive machine learning models (RF and SL) and a simple LME are considered as candidate prediction models for post‐baseline ALSFRS‐R scores. Specifically, we evaluated goodness‐of‐fit measures and how much the model predictions could improve statistical power in detecting a treatment effect using simulated clinical trials. We were able to identify the best model and describe the corresponding clinical trial scenarios where it did make a meaningful difference in statistical power.

Simulation studies suggested that using ALSFRS‐R predictions provided by RF and SL as a time varying covariate can increase the effective sample size under moderate treatment effect. They also showed that SL was somewhat more sensitive when a treatment effect exists, as evidenced by producing smaller *P*‐values on average (win rates), while still controlling type I error at a similar level.

One drawback of our prediction models is that RF and SL both do not account for within‐patient correlation. Although the LME is more interpretable and does incorporate within‐patient correlation over time, it sacrifices a small amount of predictive accuracy. A possible remedy would be to use a semiparametric LME model for the predictions.^18^ However, model complexity would increase, and interpretation could be more difficult.

Our results are similar to what has been published previously in terms of model goodness‐of‐fit but serve to highlight the importance of missing data handling as well as identifying specific scenarios, in terms of trial duration and underlying treatment effect, where predictive modeling does and does not make a meaningful difference toward the primary analysis. Furthermore, while RF was able to marginally outperform the LME model, an even more complex model (SL) was unable to explain additional variability, indicating a relative lack of significant complex nonlinear structure in currently available ALS clinical trials data types.

## Author Contributions

N.Z.: drafting/revising the manuscript, study concept and design, analysis and interpretation of data, acquisition of data, and statistical analysis; P.M.: drafting/revising the manuscript, study concept and design, analysis and interpretation of data, acquisition of data, and statistical analysis.

## Conflict of Interest

N.Z.: Paid intern at Genentech, Inc. and current graduate student at the University of Michigan. P.M.: Employee of Genentech, Inc., shareholder of F. Hoffmann La Roche, Ltd.

## Supporting information


**Figure S1.** Summary of demographic predictor variables in complete case PRO‐ACT (*n* = 552), imputed PRO‐ACT (*n* = 3160), and olesoxime (*n* = 509) datasets at baseline.
